# Impact of Knowledge Dissemination on Employee-Based Brand Equity: Mediating Role of Brand Identification and Emotional Attachment

**DOI:** 10.3389/fpsyg.2022.924139

**Published:** 2022-06-14

**Authors:** Han Liu

**Affiliations:** Department of Art and Design, School of Art, Jiangxi University of Finance and Economics, Nanchang, China

**Keywords:** internal knowledge dissemination, brand identification, emotional attachment, employee-based brand equity, organization

## Abstract

The challenging competitive situation in the market forces the organizations to recognize the crucial role of branding. Many studies focused on financial and customer perspectives and ignored the importance of employee-based brand building in the organization. Employee-based brand equity plays a vital role in increasing organizational performance. Hence, this study puts effort into brand-building and recognized many factors that develop employee-based brand equity for organizations. This study examines the role of internal knowledge dissemination and employees-based brand equity through brand identification and emotional attachment. This study also assesses the direct relationship between internal knowledge dissemination and brand identification, internal knowledge dissemination and emotional attachment, brand identification and employee-based brand equity, and emotional attachment and employee-based brand equity. For this purpose, this study adopts a convenient sampling technique and questionnaire survey method and gathered data with 712 sample sizes from employees of various clothing brands in China. For empirical examination of the data, this study considers the partial least square structural equation modeling technique and analyzed data using the Smart PLS 3.3.3 software. The outcomes revealed that internal knowledge dissemination negatively influences employee-based brand equity. This study finds a positive direct association between internal knowledge dissemination and brand identification, internal knowledge dissemination and emotional attachment, brand identification and employee-based brand equity, and emotional attachment and employee-based brand equity. Moreover, this study finds that emotional attachment and brand identification positively mediate the relationship between internal knowledge dissemination and employee-based brand equity. The findings of this study provide an insight to the organizations that effective dissemination of the internal knowledge enhances employees’ brand identification and their emotional attachments. Consequently, these positive attributes of employees play a constructive role in creating employee-based brand equity. This study also has some valuable theoretical and practical implications.

## Introduction

In this ever-changing era, it is very challenging for the organization to deal with the market dynamics to create a solid competitive position (Iglesias et al., 2019). Firms are trying to find tools and techniques to create a competitive edge. However, Hasni et al. (2018) pointed out that organizations are now focusing on branding and using it as a powerful tool for maintaining sustainability. In the preceding decades, organizations mainly emphasize branding from the consumers’ perspective, but currently, this trend is fading (Iglesias et al., 2019). In parallel with consumers’ perspective of branding, organizations are now giving attention to employee perspective as well. King and Grace (2010) also acknowledged the importance of branding with employees’ perspectives and said that the intellectual abilities of employees are the valuable asset of organization for effective branding.

The organizational efforts invested in the form of branding could pave the way for the creation of organizational brand equity (Fernández-Ruano et al., 2022). Furthermore, they stated that effective organizational brand equity is a constructive indicator for the long-term sustainability of a firm. According to the Feldwick’s (1996) point of view, brand equity could be termed as the value addition effort of the firm to generate a long-term relationship with stakeholders. Brand equity has a crucial role in achieving organizational goals and objectives in the form of increasing firm performance. Higher brand equity enables organizations to win greater trust and commitment of customers (Hanaysha and Al-Shaikh, 2021). King and Grace (2009) pointed out three essential dimensions of brand equity, namely, financial-based, consumer-based, and employee-based brand equity (EBBE). Furthermore, they revealed that the literature has pieces of evidence of brand equity creation from the perspective of financial and consumers, but there is a dearth of literature on the employee perspective.

Iglesias et al. (2019) further shed light on EBBE and said that organizations should focus on employees’ training and development to understand brand values because they can play a crucial role in making or breaking the brand. Moreover, Hanaysha and Al-Shaikh (2021) also pointed out the importance of EBBE and said that organizational performance is greatly influenced by employee contribution in the creation of the brand equity process. In addition, it is more convenient for the firm to achieve expected organizational success when there is a positive alignment between employees’ values and the brand. Scholars documented the importance in the literature of brand equity and said that firms can improve the ability of delivering promises accurately by considering EBBE as an important aspect of organizational strategy (Piehler et al., 2016; Boukis and Christodoulides, 2020; Hanaysha and Al-Shaikh, 2021). However, Vallaster and Lindgreen (2011) acknowledged that when organizations consider employees as part of brand-building strategies, they admit the importance of employees in the firm’s success. This positive signal of the organization can create a sense of emotional attachment and psychological commitment of employees to the brand.

Patwardhan and Balasubramanian (2011) noticed that employees’ emotional attachment seeks huge attention from researchers due to its important role in the brand equity process. EBBE is positively influenced by the psychological and emotional attachment of employees. These positive attributions of employees assist the organization in gaining expected outcomes (Poulis and Wisker, 2016). The employees with emotional attachment feel like an important part of organizations, and they serve their energies more efficiently in the building process of brand equity. This type of emotional bond between employees plays a favorable role in the firm’s overall performance. King and Grace (2010) shed further light and said that organizational sustainability also has a strong link with employees’ emotional attachment. However, Boukis and Christodoulides (2020) revealed that although emotional engagement is important for the creation of EBBE, there is a need to consider another important aspect of the EBBE creation process, such as brand identification.

Kuenzel and Halliday (2008) noticed that the literature argues the brand identification from the customer-brand perspective and ignores this concept from other internal stakeholders’ points of view. In addition, brand identification consequently affects the attitude and behavior of employees. Boukis and Christodoulides (2020) shed further light and said that organizational citizenship behavior could be a possible antecedent of employee brand identification. When employees feel the similarity between the brand value and their values, they feel a sense of belongingness with the brand (Lashley, 1999). Helm et al. (2016) argued in favor of employee brand identification and said that the brand equity creation process is interlinked with the brand identification of employees. Boukis and Christodoulides (2020) further argued that brand identification and internal knowledge dissemination are important aspects of the EBBE building process.

Erkmen (2018) pointed out that the internal dissemination of knowledge is the basis of the EBBE creation process. Organizational effective strategies for internal knowledge dissemination are an important tool to improve the overall organizational performance. Knowledge dissemination is a process of transferring the brand’s values to employees to deliver the organizational promise to consumers accurately (King and Grace, 2010). In addition, internal organizational efforts in knowledge dissemination favorably influenced the employee level of commitment and satisfaction. Billy and To (2013) stated that internal knowledge dissemination affects the work attitude of an employee and their long-term retention with the organization.

This study attempts to contribute to the literature in eight ways. First, this study serves the literature by adding insight into the building process of EBBE. This study tries to point out the importance of brand equity for organizations from employees’ perspectives as scholars acknowledged that there is a dearth of literature on EBBE (King and Grace, 2010; King and So, 2015; Boukis and Christodoulides, 2020). Second, based on social exchange theory, this study tries to find out how internal knowledge dissemination plays a role in the EBBE creation process. However, Erkmen (2018) documented in the literature that organizations’ internal knowledge dissemination efforts have a positive association with EBBE. Third, based on identity theory (Tajfel, 1978), this study attempts to determine the role of internal knowledge dissemination in employee brand identification activities. Boukis and Christodoulides (2020) pointed out that internal knowledge dissemination acts as a precursor to employee brand identification. Fourth, on the basis of signaling theory, this study tries to reveal the role of internal knowledge dissemination in the emotional attachment of employees. Fifth, this study tries to know how brand identification participates in EBBE, as Boukis and Christodoulides (2020) argued the importance of brand identification in the brand equity building process from an employee’s perspective. Sixth, this study also attempts to find out the positive direct relationship of emotional attachment with EBBE. Seventh, this study tries to determine the mediating role of brand identification between internal knowledge dissemination and EBBE. This study also attempts to find out the mediating role of emotional attachment between knowledge dissemination and EBBE.

The remainder of this article is structured as follows: first, this study introduces the key concept of the theoretical framework and reviews the literature for hypothesis development. Next, the methodology of the article is documented, and the findings are discussed. Next, this article presents the discussion part. Finally, this study finishes with concluding remarks as well as future research directions and limitations of the study.

## Literature Review

### Employee-Based Brand Equity

In this era of competition, organizations have to deal with a complex business marketplace (Poulis and Wisker, 2016). Moreover, organizations have to manage their tangible and intangible equities to compete with the dynamics of the market. However, according to the King and Grace’s (2010) point of view, intangible equities have more weight for competitive advantage than tangible equities. Additionally, Hasni et al. (2018) pointed out that to compete in a complex marketplace, organizations use branding as a powerful tool to maintain their sustainability. These brand-building activities of firms help them out in achieving the goals of brand equity creation (Fernández-Ruano et al., 2022).

Prados-Peña and Del Barrio-García (2021) shed light on the significance of brand equity and said that it is an important and valuable intangible asset of organizations. King and Grace (2006) pointed out that financial, customer, and employee-based brand equities are three fundamental approaches to measuring brand equity. Furthermore, they give arguments that literature gives more attention to financial and customer perceptive of brand equity and ignores the importance of EBBE. Lee et al. (2019) termed EBBE as the employee’s constructive and productive brand behaviors that are interlinked with brand identity and brand knowledge. Wilden et al. (2006) acknowledged that firms are now realizing the importance of the knowledge and skills of employees in organizational success and their constructive role in brand-building activities. Moreover, Lee et al. (2019) also commented that employees are the firm’s important stakeholders and are also termed as the organization’s internal customers in the literature. Furthermore, they acknowledged that positive internal dissemination of knowledge and organizational brand-building strategies shape the attitude and behaviors of the employee for the creation of brand equity. However, scholars noticed that the frontline employees (leaders) have more considerable role in shaping attitude and behaviors of other employees, as well as other external stakeholders in the brand-building process (Vallaster and Lindgreen, 2011).

Poulis and Wisker (2016) stated that “brand endorsement, brand-consistent behaviors, and brand allegiance” are three important dimensions of EBBE. Furthermore, they elaborated on the term brand endorsement and said that employees are willing and enthusiastic to say positive things about the brand. The brand’s positive image in employees’ minds is a favorable indication for organizations to have a long-term relationship with employees. The second important dimension is brand-consistent behaviors, which could be termed as the extent of employee perception about the uniformity of values of employees and organizations (King and So, 2015). When employees feel identification with the values of organizations, they will ultimately put their efforts into fulfilling the expectations of customers. Poulis and Wisker (2016) defined the third dimension of EBBE and stated that brand allegiance is employees’ intention to remain the part of the firm for a long time. Employee-organization long-term relationships are a positive signal for the firm in the EBBE building process.

### Internal Knowledge Dissemination

King and Grace (2010) pointed out that organizations need an effective system to provide clear direction to shape employees’ attitude and behavior for the brand equity process. Furthermore, they instructed that it is the organizational responsibility to generate required information and transfer to employees in a respectful and relevant manner. The effective internal communication system of the organization plays a central role in improving employees’ brand-building efforts (Men, 2014). Moreover, Robson and Tourish (2005) stated that organizations have to put their efforts into internal knowledge dissemination for more effective results from employees in the brand-building process. King and Grace (2010) defined knowledge dissemination as the extent of employees’ perception of the effective transformation of brand-related knowledge from organization to employee. Internal knowledge dissemination activities of organizations are concerned with transferring the knowledge to employees to appropriately satisfy consumers’ needs (King and So, 2015). Furthermore, they argued that organizations have to be serious about effective planning between information generation and communication to employees for brand equity creation.

Knowledge dissemination provides assistance to employees in understanding the rationale behind organizational strategies and guides them to deliver services based on expectations (King and Grace, 2010). Van der Bij et al. (2003) also acknowledged the importance of knowledge dissemination for organizational strategic planning. Organizations can offer a timeline of possible solutions to deal with internal and external challenges through effective dissemination of information.

Kingston (2012) documented that there are two important dimensions of knowledge dissemination with four different approaches. Moreover, the first dimension contained formal and informal approaches, and the second dimension consisted of “connect” and “collect” approaches. Using formal knowledge dissemination approach, the author performed knowledge dissemination activities within a well-defined structure or set of rules (Billy and To, 2013). In addition, the informal knowledge dissemination approach means the organizational activities for dissemination that are non-structured, and there is no hard and fast set of rules. Kingston (2012) defined the collect approach of knowledge dissemination and said that this is the approach by which the knowledge is assembled and stored in a repository. Furthermore, the author argued that the direct transformation of knowledge between organizations and employees is accounted for under the connect approach of knowledge dissemination. Bharadwaj (2014) also commented on the importance of internal knowledge dissemination and pointed out that internal knowledge dissemination has two main types, namely, as horizontal and vertical dissemination. Furthermore, they elaborated on the horizontal dissemination of knowledge and said that it occurred when employees who kept similar organizational positions transformed the knowledge between each other. Vertical knowledge dissemination occurs either in downward position (from top to bottom) or upward position (from bottom to top) (Stevanović and Gmitrović, 2015).

The above literature on internal knowledge dissemination highlights the vital role of knowledge dissemination in the improvement of employee productivity and organizational performance. The effective internal knowledge dissemination activities of organizations increase the chances of employees’ commitment and engagement. Based on the social exchange theory, this study assumes that organizations’ internal knowledge dissemination efforts increase EBBE. Employees show positive behavior on a reciprocity basis when organizations put their energies into effective knowledge dissemination. Erkmen (2018) also admitted the positive role of knowledge dissemination in creation of EBBE. Based on the literature, this study hypothesizes that:

**H1:** *Internal knowledge dissemination has a positive association with employee-based brand equity.*

### Emotional Attachment

Burris et al. (2008) noticed that employees’ psychological and emotional bond with the values and objectives of organizations is a positive indication of their long-term relationship. Furthermore, they stated that this emotional tie between employees and organizations plays a significant role in achieving the firm’s desired goals. Poulis and Wisker (2016) stated that when employees feel identified with organizational values, they put their efforts more enthusiastically into brand management activities. Furthermore, they argued that EBBE is one of the possible outcomes of employee emotional and psychological attachment. Additionally, King and Grace (2010) revealed that these positive attributes of employees have considerable worth in the building process of EBBE.

Hang et al. (2020) stated that emotional attachments could make significant attitudinal and behavioral changes in the stimulus. The emotional attachment of employees rewards the organization with two valuable employee attributes in the form of brand attitude and brand loyalty (Patwardhan and Balasubramanian, 2011). Grisaffe and Nguyen (2011) also argued in favor of brand loyalty as the significant consequence of emotional attachment. Furthermore, they stated that emotional attachment is a psychological state deeply rooted in brand involvement, brand attitude, and brand satisfaction. King and Grace (2012) further shed light on important attitudes and behaviors of employees and point out brand commitment and organizational citizenship behavior as important antecedents of employee emotional attachment. Further, they point out three important aspects that can affect the internal brand building management from the employees’ perspective. First, organizational socialization is the extent of employees’ belief to which they perceive that the overall strategies and environment of the organization are suitable for them to learn values and expectations related to the brand. Piehler et al. (2016) also agreed with the importance of organizational socialization and said that effective internal transformation of organizational brand-building strategies is a valuable organizational attempt for EBBE. Second, relationship orientation could be defined as the degree of positive organizational intentions for retaining the long-term relationships with their employees. Third, employee receptiveness is the extent to employee willingness or readiness to receive the organizational brand-related information, which, in turn, affects their brand-related attitude and behavior (King and Grace, 2012).

Patwardhan and Balasubramanian (2011) acknowledged that “pleasure, arousal, and dominance” are three important dimensions of emotional attachment. Furthermore, they defined the term pleasure and said that it is the extent of stimulus positive emotional state of joy, which he or she feels with some particular brand. Moreover, arousal is the stimulus tendency to encourage others’ attitudes in a positive way about the brand. The term dominance describes the extent to which a stimulus feels that the brand becomes an essential part of his or her lifestyle (Patwardhan and Balasubramanian, 2011).

The above-discussed literature underlines the importance of emotional attachment role in increasing the performance of employees and the organization. On the basis of signaling theory, this study tries to find out the role of internal knowledge dissemination in the emotional attachment of employees. Organizations’ effective internal knowledge dissemination activities are a constructive signal toward employees to enhance their attachment to the brand. Hang et al. (2020) also acknowledged that organizations’ communication strategies pave the way for employees’ strong bonds with their organization. On the basis of attachment theory, these positive attributes of emotional attachment shape employees’ behavior during the creation process of EBBE. Furthermore, this study attempts to check the mediating role of emotional attachment between knowledge dissemination and EBBE. Based on the literature, this study hypothesizes that:

**H2:** *Internal knowledge dissemination has a positive association with emotional attachment.*

**H3:** *Emotional attachment has a positive association with employee-based brand equity.*

**H4:** *Emotional attachment mediates the relationship between internal knowledge dissemination and employee-based brand equity.*

### Brand Identification

Vallaster and Lindgreen (2011) identified that efficient delivery of brand promise is employees’ responsibility because they have a crucial role in internal brand management strategies. Furthermore, they argued that organizations have to put effort into managing positive employees’ attitudes and behaviors for brand-building activities. Employees are the key source for organizations to create a competitive advantage and maintain their sustainability (Piehler et al., 2016). Furthermore, they pointed out that employees’ brand identification is an important factor in the EBBE building process. Hassan (2012) defined brand identification as an imperative psychological state of employees that reflects their attachment to the workplace, which influences positively on job-related outcomes. In addition, Piehler et al. (2016) commented that for efficient and effective delivery of brand promise, it is necessary for employees to thoroughly understand the purpose and values of the brand. Furthermore, they stated that without such cognitive recognition, employees cannot participate with their full energies in brand-building process.

According to Kuenzel and Halliday (2008), “prestige, satisfaction, and corporate communications” are three important determinants of brand identification. By prestige, the author means the positive perceptions and opinions of other people about the brand. Furthermore, these positive brand evaluations of other people endorse the thoughts of employees in a positive manner for the creation of brand identification. Satisfaction indicates the emotional and cognitive response of other people toward the brand after purchasing and using it. Moreover, this positive response from people about the brand also helps out employees in brand identification. Corporate communication is the extent to which firms share knowledge in formal or informal ways with each other. This positive management instrument paints a positive picture of the organization in employees’ minds and has a positive effect on brand identification (Kuenzel and Halliday, 2008).

Hassan (2012) identified “cognitive, affective, and evaluation” as three important components of brand identification. Moreover, cognitive identification indicated the degree to which people related themselves in relation to the organization. Affective identification refers to the extent to which people feel an essential part of a particular organization (Piehler et al., 2016). The evaluative component refers to the extent of employees’ evaluation of the brand image and their assessment of the value of organizations (Hassan, 2012). The internal brand management efforts of the organization can play a considerable role in the creation of these three components of brand identification (Piehler et al., 2016).

**FIGURE 1:**
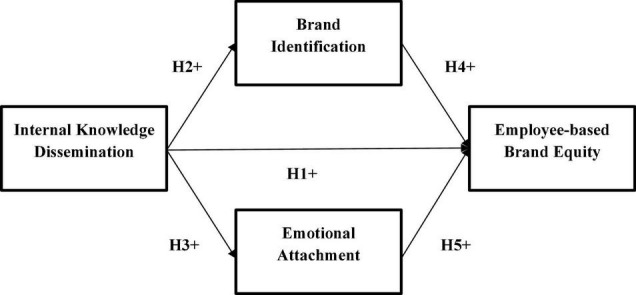
Conceptual framework.

On the basis of identity theory, this study assumes that employee brand identification increase when organizations effectively disseminate brand-related knowledge to their employees. Boukis and Christodoulides (2020) also agreed that effective brand-related knowledge dissemination enhances the brand identification of employees. In addition, this study also attempts to check the influence of employees’ brand identification on EBBE. This study also tries to determine the mediating role of brand identification between knowledge dissemination and EBBE. For empirical investigation of these assumptions, Figure 1 explains this study’s research model and thus hypotheses that:

**H5:** *Internal knowledge dissemination has a positive association with brand identification.*

**H6:** *Brand identification has a positive association with employee-based brand equity.*

**H7:** *Brand identification mediates the relationship between internal knowledge dissemination and Employee-based brand equity.*

## Research Methods

### Study Design

This study applies a convenient sampling technique to collect data for empirical examination of this study model. The data were gathered from employees of top clothing brands in China. To shortlist the top clothing brands, the author first developed a mini questionnaire based on famous top clothing brands and distributed it among 30 male and 30 female final-year students of BBA marketing and asked them which clothing brand they understand as the top. The author also asked for suggestions from respondents regarding other top clothing brand names that the author missed, and according to respondents, these should also be included in this questionnaire. In this way, the author also included other clothing brand names. Second, the author also consulted the results of mini questionnaires with senior researchers, and they acknowledged mini questionnaires’ outcomes. In this way, the author shortlisted the top well-known brands. The author personally visited the targeted clothing brands and got permission from their managers to address his academic purpose. The author convinced managers by assuring that the data would be utilized only for academic objectives.

Moreover, the author also ensured the managers that the outcomes and practical implications of this research would be shared with them upon their request, and thus most of the managers showed their consent. At the request of the author, managers shared the details of employees. The author targeted 3,000 employees and personally distributed questionnaires among these employees. The questionnaire was developed based on a cover letter. The cover letter explained the objective of the data collection and trusted the employees about their data confidentiality. Moreover, the cover letter also ensures the employees that their answers are not good or bad: “Instead, your actual answer will be considered good, so don’t consult with your colleagues for the answer.” This step surely boosts up the confidence of the employees so that they fill questionnaires without any hesitation but with their free will. Furthermore, the author adopted a time lag data approach for gathering the data and decided to distribute questionnaires in three turns to reduce the common method bias. Thus, the author designed questionnaires to recognize the same respondents based on hidden codes.

In the first turn, the author distributed demographics and IV- (interdissemination knowledge) related questionnaires and collected 1,919 valid questionnaires out of 3,000. After a 45 days gap, the author distributed questionnaires based on mediator variables (brand identification and emotional attachment). In this second round, the author collected 899 complete questionnaires. After an additional 45 days gap, the author again distributed questionnaires based on DV (EBBE), and in this third round, the author gathered 726 responses. Out of 726 responses, the author found 14 incomplete responses. In this scenario, this study collects 712 complete and valid responses. Hence, the empirical outcomes of this study were based on the 712 sample size.

### Measures

The study measures respondents’ responses by using 5-point Likert scale. This scale consists of points 1 to 5, where 1 represents “strongly disagree,” 2 represents “disagree,” 3 represents “neutral,” 4 represents “agree,” and 5 represents “strongly agree.” Previously validated items were adopted to measure this study model’s variables. This study measures internal knowledge dissemination with the seven items developed by King and Grace (2010). The sample item includes “The information provided to me when I was employed helped me to understand my role in the context of what the organization is trying to achieve.” The variable brand identification was measured with 4 items developed by Boukis and Christodoulides (2020). The sample item includes “My sense of pride toward (my company) brand is reinforced by the brand-related messages.” The variable emotional attachment was measured through nine items developed by Patwardhan and Balasubramanian (2011). The sample item includes, “Sometimes I feel I can’t control my thoughts as they are obsessively on this brand.” The variable EBBE was measured through five items developed by Boukis and Christodoulides (2020). The sample item includes “I always consider the impact on the company’s brand when I make decisions.”

### Demographic Information

This study also assesses the demographic information of the participants. The total number of participants was 712. Out of this, 380 were females, and 332 were males. A total of 180 participants were 20–30 years old, 263 were 31–40 years old, and 141 were 41–50 years old; 51 were above 50 years old; 48 participants have 10–12 years of education; 135 have bachelor’s education; 291 have master’s education, and 238 have other professional kinds of education. A total of 119 respondents have less than 2 years of experience, 167 have 2–4 years of experience, 233 have 5–7 years of experience, 145 have 8–10 years of experience, and 48 have 11 years and more than 11 years of experience (see Table 1).

**TABLE 1:** Demographic information.

Categories	Subcategories	Numbers	Percentage
Gender	Male	332	46.6
	Female	380	53.4
Age	20–30 years	180	25.3
	31–40 years	263	36.9
	41–50 years	141	19.8
	51 years or above	128	18.0
Education	Matric to intermediate	48	6.7
	Bachelor	135	19.0
	Master	291	40.9
	Any other	238	33.4
Experience	Less than 2 years	119	16.7
	2 to 4 years	167	23.5
	5 to 7 years	233	32.7
	8 to 10 years	145	20.4
	11 years onward	48	6.7

### Results

This study considers the structural equation modeling (SEM) approach by utilizing partial least squares (PLS) to analyze the data. PLS-SEM is a variance-based approach; however, it is different from a covariance-based approach like AMOS (Nawaz et al., 2022), and the main reason behind its adoption is because it is convenient for both confirmatory and exploratory studies (Avotra et al., 2021). SEM depends on covariance-based (CB-SEM) and PLS-SEM methods (Hair et al., 2014). The key variation in both methods is that CB-SEM is useful for accepting and rejecting the theories; conversely, PLS-SEM is useful for advancing and extending the theories (Yingfei et al., 2021). Thus, this study’s data were analyzed through the Smart PLS 3.3.3 software. The Smart PLS provides outcomes in two parts: the first part is measurement and the second part is the structural path. Smart PLS also efficiently handles complex or even short sample sizes data analysis.

The measurement of the research framework is based on two parts, namely, reliability and validity. The reliability of the framework mainly depends on standard values of Cronbach’s alpha, roh-A, composite reliability, and average variance extract (Hair et al., 2014, 2016). This study’s reliabilities of all variables are presented in Table 2. As per the criteria, the acceptable Cronbach’s alpha value is up to or above 0.7 (Hair et al., 2019). This study’s research framework variables and Cronbach’s alpha values are based on the given criteria. Such as the IV (knowledge dissemination), mediators (brand identification and emotional attachment), and DV (EBBE), Cronbach’s alpha values are 0.819, 0.921, 0.867, and 0.914, respectively. However, Cronbach’s alpha values are as per the given criteria and thus accepted. The roh-A values of all variables are based on the given criteria. Similar to Cronbach’s alpha, the composite reliability criteria are also up to or above 0.7. All variables’ composite reliability is also more than 0.7. Thus, this reliability is also accepted. Finally, the average variance extract (AVE) value is acceptable if it is above 0.5. This study models all variables’ AVE values are more than 0.5. Thus, this reliability is also accepted.

**TABLE 2:** Reliability and validity of the study constructs.

Construct	Item	Outer loadings	VIF	Alpha	roh-A	Composite reliability	AVE
BI	BI1	0.760	1.558	0.819	0.821	0.881	0.650
	BI2	0.799	1.771				
	BI3	0.848	2.279				
	BI4	0.815	2.059				
EA	EA1	0.720	1.847	0.921	0.923	0.934	0.612
	EA2	0.811	2.763				
	EA3	0.790	2.831				
	EA4	0.821	3.172				
	EA5	0.804	2.852				
	EA6	0.845	4.063				
	EA7	0.761	2.391				
	EA8	0.781	2.401				
	EA9	0.698	1.626				
EBBE	EBBE1	0.839	1.774	0.867	0.896	0.907	0.710
	EBBE2	0.872	2.364				
	EBBE3	0.855	2.647				
	EBBE4	0.802	2.360				
IKD	IKD1	0.795	2.045	0.914	0.917	0.932	0.663
	IKD2	0.863	3.033				
	IKD3	0.858	2.950				
	IKD4	0.850	2.889				
	IKD5	0.878	3.334				
	IKD6	0.706	1.615				
	IKD7	0.734	1.757				

*BI, brand identification; EA, emotional attachment; EBBE, employee-based brand equity; IKD, internal knowledge dissemination.*

**FIGURE 2:**
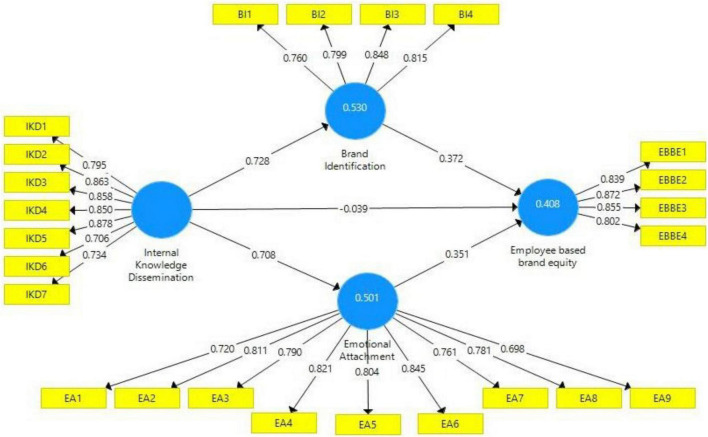
Path estimates.

Furthermore, Table 2 also presents the outer loadings of all variable items. Values of 0.7 or above 0.7 are considered acceptable for outer loadings (Xiaolong et al., 2021). The items’ outer loadings values in this study model are above 0.7 except for two items (Figure 2), one item EA9 of mediator variable (emotional attachment). The item EA9 is retained in the model because it is close to 0.7 and does not affect overall model reliability. The second item, EBBE5 of DV (EBBE), was deleted because of lower outer loading.

Table 2 also explains all variable items’ variance inflation factor (VIF) values. The purpose of VIF measurement is to examine the collinearity issue in the model. A VIF value less than 0.5 is considered a fit for the model (Hair et al., 2019). In this study, all variables’ items VIF values are in the range of 1.558–3.334. Hence, it is entrusted that the model of this study is free from collinearity issues.

According to Hair et al. (2019), the R^2^ value of latent variables up to 0.5 shows moderate strength in the model. This study constructs (brand identification, emotional attachment, and EBBE) R^2^ values as 0530, 0.501, and 0.408, respectively. Thus, based on these R^2^ values, this study model shows moderate strength. Finally, this study’s latent variables also have Q^2^ values more than zero. It also revealed this study model’s significance.

Fornell-Larcker criterion and heterotrait-monotrait (HTMT) were applied to check the model discriminant validity in this study (Hair et al., 2016). As per the Fornell-Larker criterion, all constructs’ square root of average variance extract values was taken (Fornell and Larcker, 2016; Hair et al., 2016). This study models values of the Fornell-Larker criterion explained in Table 3. According to the criteria, all the above values of each column mentioned in Table 3 should be greater than their lower values in the same column. Hence, this study model fulfills the Fornell-Larker criterion by achieving discriminant validity (Fornell and Larcker, 2016; Hair et al., 2016). As per the HTMT criterion, all construct of models values should be below 0.85 (Hair et al., 2016). This study models construct values of HTMT are presented in Table 4, and these values are based on the given threshold as all values are below 0.85. Hence, discriminant validity is appropriate for this study.

**TABLE 3:** Discriminant validity (Fornell-Larker-1981 criteria).

Constructs	BI	EA	EBBE	IKD
BI	**0.806**			
EA	0.708	**0.783**		
EBBE	0.593	0.587	**0.843**	
IKD	0.728	0.708	0.481	**0.814**

*BI, brand identification; EA, emotional attachment; EBBE, employee based brand equity; IKD, internal knowledge dissemination. The bold values indicate the significance.*

**TABLE 4:** Discriminant validity (HTMT).

Constructs	BI	EA	EBBE	IKD
BI	–	–	–	–
EA	0.806	–	–	–
EBBE	0.669	0.622	–	–
IKD	0.841	0.762	0.521	–

*BI, brand identification; EA, emotional attachment; EBBE, employee-based brand equity; IKD, internal knowledge dissemination.*

### Hypotheses Testing

The empirical analysis of this study hypotheses was examined through 5,000 samples of bootstrapping techniques for statistical outcomes, and Table 5 explains the total direct, indirect, and path outcomes of this study model (Hair et al., 2014, 2016). This study’s hypotheses’ statistical acceptance and rejection are established on *t*- and *p*-values (Hair et al., 2014). Table 6 explains the outcomes of this study’s hypotheses. H1 of this study predicted the positive direct association between knowledge dissemination and EBBE. Moreover, statistical outcomes (*t* = 0.807, *p* = 0.419) revealed their negative direct association. Hence, as per outcomes, knowledge dissemination negatively influences the EBBE. Thus, H1 is rejected. H2 of this study observed a positive direct relationship between knowledge dissemination and brand identification. According to the statistical outcome (*t* = 26.050, *p* = 0.000), this study confirms that internal knowledge dissemination positively influences brand identification as the path coefficient value reveals that one unit change in internal knowledge dissemination would bring a change of 0.728 in brand identification. Hence, H2 is accepted. H3 of this study expected a positive direct association between internal knowledge dissemination and emotional attachment. The statistical outcomes (*t* = 24.267, *p* = 0.000) reveal that internal knowledge dissemination positively influences the emotional attachment. As per the beta value, one unit variation in internal knowledge dissemination would lead 0.708 variation in emotional attachment. Thus, H3 is accepted. H4 of this study predicted a positive relationship between brand identification and EBBE. The statistical results (*t* = 6.405, *p* = 0.000) explain that brand identification positively influences the EBBE such as according to the path coefficient value; one unit variation in brand identification would bring 0.372 variation in EBBE. Thus, H4 is accepted. H5 of this study expected a positive association between emotional attachment and EBBE and as per statistical outcomes (*t* = 7.714, *p* = 0.000); it is confirmed that emotional attachment positively influences the EBBE. The beta value shed light that one unit variation in emotional attachment would lead to 0.351 variation in EBBE. Hence, H5 is accepted.

**TABLE 5:** Direct, indirect, and total path estimates.

Direct path	Beta	SD	*t*	*p*
BI - > EBBE	0.372	0.058	6.405	0.000
EA - > EBBE	0.351	0.046	7.714	0.000
IKD - > BI	0.728	0.028	26.050	0.000
IKD - > EA	0.708	0.029	24.267	0.000
IKD - > EBBE	−0.039	0.048	0.807	0.419
**Indirect Path**				
IKD - > BI - > EBBE	0.271	0.043	6.291	0.000
IKD - > EA - > EBBE	0.249	0.034	7.352	0.000
**Total Path**				
BI - > EBBE	0.372	0.058	6.405	0.000
EA - > EBBE	0.351	0.046	7.714	0.000
IKD - > BI	0.728	0.028	26.050	0.000
IKD - > EA	0.708	0.029	24.267	0.000
IKD - > EBBE	0.481	0.040	11.883	0.000

*BI, brand identification; EA, emotional attachment; EBBE, employee-based brand equity; IKD, internal knowledge dissemination.*

**TABLE 6:** Hypotheses testing.

Hypotheses		Coefficient (Beta)	S.D	*t*	*p*	Status
H1	IKD - > EBBE	−0.039	0.048	0.807	0.419	Not supported
H2	IKD - > BI	0.728	0.028	26.050	0.000	Supported
H3	IKD - > EA	0.708	0.029	24.267	0.000	Supported
H4	BI - > EBBE	0.372	0.058	6.405	0.000	Supported
H5	EA - > EBBE	0.351	0.046	7.714	0.000	Supported
**Mediation hypotheses**		**Coefficient (Beta)**	**S.D**	***t***	***p***	**Status**
H6	IKD - > BI - > EBBE	0.271	0.043	6.291	0.000	Supported
H7	IKD - > EA - > EBBE	0.249	0.034	7.352	0.000	Supported

*BI, brand identification; EA, emotional attachment; EBBE, employee-based brand equity; IKD, internal knowledge dissemination.*

Moreover, the H6 and H7 expected mediation role of brand identification and emotional attachment between the association of internal knowledge dissemination and EBBE. The statistical values (*t* = 6.291, *p* = 0.000) of H6 reveal that brand identification positively mediated the relationship between internal knowledge dissemination and EBBE. Hence, H6 is accepted. According to the statistical values (*t* = 7.352, *p* = 0.000) of H7, it is confirmed that emotional attachment positively mediated the relationship between internal knowledge dissemination and EBBE. Thus, H7 is accepted. The outcomes of *t*-values of hypotheses H6 and H7 also revealed that emotional attachment plays the highest mediation role between internal knowledge dissemination and EBBE rather than brand identification.

## Discussion

Currently, organizations’ primary focus is to gain a competitive edge by creating brand equity. Organizations are now realizing the importance of brand equity as it creates competitive advantages and wealth for the organization (Lee et al., 2019; Liu et al., 2020; Maleki Minbashrazgah et al., 2021). EBBE is a vital construct regarding organizational brand equity, and due to its high importance, this study attempts to find out the brand-building process from employees’ perspectives. This study proposes that internal knowledge dissemination positively predicts the EBBE based on the social exchange theory. Boukis and Christodoulides (2020) argued the importance of knowledge dissemination regarding EBBE. This study also predicts that brand identification and emotional attachment mediate the relationship between internal knowledge dissemination and EBBE.

Moreover, on the basis of signaling theory, this study also assesses the direct association between internal knowledge dissemination and brand identification, internal knowledge and emotional attachment, brand identification and EBBE, and emotional attachment and EBBE. For this purpose, this study proposes seven hypotheses (Table 6). First, H1 of this study predicted a positive association between internal knowledge dissemination and EBBE, but this study finds that internal knowledge dissemination negatively influences the EBBE. Hence, H1 was not as per the prediction of this study, thus rejected. Second, H2 of this study has been developed to explore the relationship between internal knowledge dissemination and brand identification. The outcomes revealed that internal brand dissemination positively impacts brand identification. Third, H4 predicted the direct positive association between brand identification and EBBE. The outcomes of this study were based on the prediction, such as brand identification positively influencing the EBBE. Fourth, the H3 of this study predicted a positive association between internal knowledge dissemination and emotional attachment. The results of this study were aligned with the prediction that internal knowledge dissemination has a positive impact on emotional attachment. Fifth, H5 of this study expected a positive relationship between emotional attachment and EBBE.

The outcomes of this study revealed that emotional attachment positively influences the EBBE. Hence, H5 outcomes were also based on the prediction. Moreover, this study also assesses the mediating role of brand identification and emotional attachment between the association of internal knowledge dissemination and EBBE. For this purpose, this study examines H6 and H7, and the results revealed that brand identification and emotional attachment positively mediated the relationship between internal knowledge dissemination and EBBE. Hence, the outcomes of this study, H5 and H6, were based on the prediction. Even though this study finds a negative association between internal knowledge dissemination and EBBE, this study’s outcomes revealed the importance of brand identification and employees’ emotional attachment. Both are significant mediators between the relationship of internal knowledge dissemination and EBBE.

### Theoretical and Practical Implications

This study has various theoretical and practical implications. This study extends the theoretical literature on EBBE in a significant way. First, this study examines the direct association between internal knowledge dissemination and EBBE and found negative consequences. This study finds that such outcomes may be due to poor management and policies. Second, this study examines the direct association of internal knowledge dissemination with brand identification and found positive outcomes such as employee brand identification increased through organizational internal knowledge dissemination. Third, this study also assesses the direct association of internal knowledge dissemination with employees’ emotional attachment and found positive outcomes such as employees’ emotional attachment to the brand positively increased by organizational internal knowledge dissemination. Hence, this study extends the literature on internal knowledge dissemination as a developer of employees’ brand identification and brand emotional attachment. Fourth, this study also extends the literature by building the relationship between brand identification and EBBE. The outcomes confirm that brand identification works as a booster of EBBE. Fifth, this study confirms that emotional attachment also enhances EBBE. Hence, this study extends the literature on emotional attachment. Moreover, this study finds emotional attachment and brand identification as mediators in the relationship between internal knowledge dissemination and EBBE. This study extends the literature on brand identification and emotional attachment as mediators between internal knowledge dissemination and EBBE. Practically, this study guides managers as this study finds internal knowledge dissemination as an essential construct for organizations. Even though its direct association with EBBE was not positive, brand identification and emotional attachment can play a role as a game-changer in building EBBE. Through organizations’ rational internal knowledge dissemination, organizations can boost employees’ brand identification and brand emotional attachment. Consequently, for the building of EBBE, organizations rationally disseminate internal knowledge among their employees. The complete and proper knowledge of the employees about their company brands creates identification and emotional attachment, which positively develops employee brand-building equity.

## Limitations

This study has limitations, which have the opportunities for further studies. First, this study collects data for empirical analysis through a questionnaire survey method; future research may collect data by applying other methods such as interviews. Second, this study applies a time lag data approach for data collection to avoid common method bias. Future research can collect data in different ways to avoid common methods bias, such as including marker variables or adding reverse coded items in variable scales. Third, this study collects data from clothing brands. Future research may enlarge the data size or adopt longitudinal data methods to confirm this study outcomes. Fourth, this study collects data from the secondary sector; the author encourages future researchers to conduct a similar study by collecting data from other sectors like primary or territory. This step also helps to verify this study outcomes. Fifth, this study does not include a moderator variable between the direct association of internal knowledge dissemination and EBBE, maybe a moderator effect turns this negative relationship into a positive relationship. Future studies may check the moderator effect of top management support, leader-member exchange, and organizational culture between internal knowledge dissemination and EBBE. Sixth, future studies may extend our study model by examining other mediators, such as brand knowledge and employee absorptive capacity. Finally, this study was conducted in China, and future research may conduct a similar study in other developing or developed countries, especially in western countries.

## Conclusion

Organizations have to face competition from their national and international competitors, and employee-based branding has a vital role in creating a competitive edge. Branding is a significant organizational tool to enhance performance and differentiate themselves in dynamic markets. Hence, organizations can achieve a competitive edge through building EBBE. For this purpose, this study develops an empirical model and examined internal knowledge dissemination’s role in promoting EBBE. This study examines the direct relationships between internal knowledge dissemination and brand identification, internal knowledge dissemination and emotional attachment, brand identification and EBBBE, and emotional attachment and EBBE. Moreover, this study also assesses a mediating role of brand identification and emotional attachment in this relationship between internal knowledge dissemination and EBBE. This study depicts that organizations, through their rational internal knowledge dissemination, can develop employees’ emotional attachment and band identification and, by building these organizations, can enhance EBBE.

## Data Availability Statement

The original contributions presented in this study are included in the article/supplementary material, further inquiries can be directed to the corresponding author.

## Ethics Statement

The studies involving human participants were reviewed and approved by the Jiangxi University of Finance and Economics, China. The patients/participants provided their written informed consent to participate in this study. This study was conducted in accordance with the Declaration of Helsinki.

## Author Contributions

HL conceived, designed the concept, and wrote the manuscript. The author read and agreed to the published version of the manuscript.

## Conflict of Interest

The author declares that the research was conducted in the absence of any commercial or financial relationships that could be construed as a potential conflict of interest.

## Publisher’s Note

All claims expressed in this article are solely those of the authors and do not necessarily represent those of their affiliated organizations, or those of the publisher, the editors and the reviewers. Any product that may be evaluated in this article, or claim that may be made by its manufacturer, is not guaranteed or endorsed by the publisher.

## References

[B1] Avotra A. A. R. N. Chenyun Y. Yongmin W. Lijuan Z. Nawaz A. (2021). Conceptualizing the state of the art of corporate social responsibility (CSR) in green construction and its nexus to sustainable development. *Front. Environ. Sci.* 9:541. 10.3389/fenvs.2021.774822

[B2] Bharadwaj A. (2014). Planning internal communication profile for organizational effectiveness. *IIM Kozhikode Soc. Manag. Rev.* 3 183–192. 10.1007/s11606-007-0124-5 17372791 PMC1829429

[B3] Billy T. W. To W. M. (2013). The effect of internal information generation and dissemination on casino employee work related behaviors. *Int. J. Hosp. Manag.* 33 475–483. 10.1016/j.ijhm.2012.11.007

[B4] Boukis A. Christodoulides G. (2020). Investigating key antecedents and outcomes of employee-based brand equity. *Eur. Manag. Rev.* 17 41–55. 10.1111/emre.12327

[B5] Burris E. R. Detert J. R. Chiaburu D. S. (2008). Quitting before leaving: the mediating effects of psychological attachment and detachment on voice. *J. Appl. Psychol.* 93:912. 10.1037/0021-9010.93.4.912 18642993

[B6] Erkmen E. (2018). Managing your brand for employees: understanding the role of organizational processes in cultivating employee brand equity. *Adm. Sci.* 8:52. 10.3390/admsci8030052

[B7] Feldwick P. (1996). What is brand equity anyway, and how do you measure it? *Mark. Res. Soc. Journal.* 38 1–17. 10.1177/147078539603800201

[B8] Fernández-Ruano M. L. Frías-Jamilena D. M. Polo-Peña A. I. Peco-Torres F. (2022). The use of gamification in environmental interpretation and its effect on customer-based destination brand equity: the moderating role of psychological distance. *J. Destin. Mark. Manag.* 23:100677. 10.1016/j.jdmm.2021.100677

[B9] Fornell C. Larcker D. F. (2016). Evaluating structural equation models with unobservable variables and measurement error. *J. Mark. Res. This* 18 39–50. 10.1177/002224378101800104

[B10] Grisaffe D. B. Nguyen H. P. (2011). Antecedents of emotional attachment to brands. *J. Bus. Res.* 64 1052–1059. 10.1016/j.jbusres.2010.11.002

[B11] Hair J. F. Risher J. J. Sarstedt M. Ringle C. M. (2019). When to use and how to report the results of PLS-SEM. *Eur. Bus. Rev.* 31 2–24. 10.1108/EBR-11-2018-0203

[B12] Hair J. F. Sarstedt M. Hopkins L. Kuppelwieser V. G. (2014). Partial least squares structural equation modeling (PLS-SEM): an emerging tool in business research. *Eur. Bus. Rev.* 26 106–121. 10.1108/EBR-10-2013-0128/FULL/HTML

[B13] Hair J. J. Hult G. Ringle C. Sarstedt M. (2016). *A PRIMer on Partial Least Squares Structural Equation Modeling (PLS-SEM).* Thousand Oaks, CA: Sage.

[B14] Hanaysha J. R. Al-Shaikh M. E. (2021). An examination of customer relationship management dimensions and employee-based brand equity: a study on ride-hailing industry in Saudi Arabia. *Res. Transp. Bus. Manag.* 100719. 10.1016/j.rtbm.2021.100719

[B15] Hang H. Aroean L. Chen Z. (2020). Building emotional attachment during COVID-19. *Ann. Tour. Res.* 83:103006. 10.1016/j.annals.2020.103006 32834221 PMC7373071

[B16] Hasni M. J. S. Salo J. Naeem H. Abbasi K. S. (2018). Impact of internal branding on customer-based brand equity with mediating effect of organizational loyalty: an empirical evidence from retail sector. *Int. J. Retail Distrib. Manag.* 46 1056–1076. 10.1108/IJRDM-07-2017-0148

[B17] Hassan S. (2012). Employee attachment to workplace: a review of organizational and occupational identification and commitment. *Int. J. Organ. Theory Behav.* 15 383–422 10.1108/IJOTB-15-03-2012-B002

[B18] Helm S. V. Renk U. Mishra A. (2016). Exploring the impact of employees’ self-concept, brand identification and brand pride on brand citizenship behaviors. *Eur. J. Mark.* 50 58–77. 10.1108/EJM-03-2014-0162

[B19] Iglesias O. Markovic S. Rialp J. (2019). How does sensory brand experience influence brand equity? Considering the roles of customer satisfaction, customer affective commitment, and employee empathy. *J. Bus. Res.* 96 343–354. 10.1016/j.jbusres.2018.05.043

[B20] King C. Grace D. (2006). Exploring managers’ perspectives of the impact of brand management strategies on employee roles within a service firm. *J. Serv. Mark.* 20 369–380 10.1108/08876040610691266

[B21] King C. Grace D. (2009). Employee based brand equity: a third perspective. *Serv. Mark. Q.* 30 122–147. 10.1080/15332960802619082

[B22] King C. Grace D. (2010). Building and measuring employee-based brand equity. *Eur. J. Mark.* 44 938–971 10.1108/03090561011047472

[B23] King C. Grace D. (2012). Examining the antecedents of positive employee brand-related attitudes and behaviours. *Eur. J. Mark.* 46 469–488 10.1108/03090561211202567

[B24] King C. So K. K. F. (2015). Enhancing hotel employees’ brand understanding and brand-building behavior in China. *J. Hosp. Tour. Res.* 39 492–516. 10.1177/1096348013491602

[B25] Kingston J. (2012). Choosing a knowledge dissemination approach. *Knowl. Process Manag.* 19 160–170. 10.1002/kpm.1391

[B26] Kuenzel S. Halliday S. V. (2008). Investigating antecedents and consequences of brand identification. *J. Prod. Brand Manag.* 17 293–304

[B27] Lashley C. (1999). Employee empowerment in services: a framework for analysis. *Pers. Rev.* 28 169–191

[B28] Lee Y.-H. Hsiao C. Chan H.-Y. Lee I.-C. (2019). Explorations of employee-based brand equity in the banking industry from a perceived-leadership perspective. *Int. J. Bank Mark.* 38 425–455

[B29] Liu A. X. Hsu C. H. Fan D. X. (2020). From brand identity to brand equity: a multilevel analysis of the organization–employee bidirectional effects in upscale hotels. *J. Contemp. Hosp. Manag.* 32 2285–2304

[B30] Maleki Minbashrazgah M. Bagheri Garbollagh H. Varmaghani M. (2021). Brand-specific transactional leadership: the effects of brand-building behaviors on employee-based brand equity in the insurance industry. *Kybernetes ahead-of-print.* 10.1108/K-03-2021-0201

[B31] Men L. R. (2014). Strategic internal communication: transformational leadership, communication channels, and employee satisfaction. *Manag. Commun. Q.* 28 264–284.

[B32] Nawaz A. Chen J. Su X. Zahid Hassan H. M. (2022). Material based penalty-cost quantification model for construction projects influencing waste management. *Front. Environ. Sci.* 10:807359. 10.3389/fenvs.2022.807359

[B33] Patwardhan H. Balasubramanian S. K. (2011). Brand romance: a complementary approach to explain emotional attachment toward brands. *J. Prod. Brand Manag.* 20 297–308

[B34] Piehler R. King C. Burmann C. Xiong L. (2016). The importance of employee brand understanding, brand identification, and brand commitment in realizing brand citizenship behaviour. *Eur. J. Mark.* 50 1575–1601

[B35] Poulis A. Wisker Z. (2016). Modeling employee-based brand equity (EBBE) and perceived environmental uncertainty (PEU) on a firm’s performance. *J. Prod. Brand Manag.* 25 490–503

[B36] Prados-Peña M. B. Del Barrio-García S. (2021). Key antecedents of brand equity in heritage brand extensions: the moderating role of tourist heritage experience. *Eur. Res. Manag. Bus. Econ.* 27:100153. 10.1016/j.iedeen.2021.100153

[B37] Robson P. J. A. Tourish D. (2005). Managing internal communication: an organizational case study. *Corp. Commun. An Int. J.* 10 213–222.

[B38] Stevanović M. I. Gmitrović A. M. (2015). Importance and role of internal communication in organizations. *Appl. Mech. Mater.* 806 302–307.

[B39] Tajfel H. E. (1978). *Differentiation Between Social Groups: Studies in the Social Psychology of Intergroup Relations.* Academic Press. Cambridge, MA

[B40] Vallaster C. Lindgreen A. (2011). Corporate brand strategy formation: brand actors and the situational context for a business-to-business brand. *Ind. Mark. Manag.* 40 1133–1143.

[B41] Van der Bij H. Michael Song X. Weggeman M. (2003). An empirical investigation into the antecedents of knowledge dissemination at the strategic business unit level. *J. Prod. Innov. Manag.* 20 163–179.

[B42] Wilden R. M. Gudergan S. Lings I. N. (2006). Employee-based brand equity. In *Proceedings of Australian and New Zealand Marketing Academy Conference (ANZMAC)*, Brisbane

[B43] Xiaolong T. Gull N. Iqbal S. Asghar M. Nawaz A. Albasher G. (2021). Exploring and validating the effects of mega projects on infrastructure development influencing sustainable environment and project management. *Front. Psychol.* 12:1251. 10.3389/fpsyg.2021.663199 33935923 PMC8085247

[B44] Yingfei Y. Mengze Z. Zeyu L. Ki-Hyung B. Avotra A. A. R. N. Nawaz A. (2021). Green logistics performance and infrastructure on service trade and environment-measuring firm’s performance and service quality. *J. King Saud Univ.* 34:101683.

